# ^18^F-FDG PET/CT metabolic parameters correlate with EIF2S2 expression status in colorectal cancer

**DOI:** 10.7150/jca.57926

**Published:** 2021-08-03

**Authors:** Jian-Wei Yang, Ling-Ling Yuan, Yan Gao, Xu-Sheng Liu, Yu-Jiao Wang, Lu-Meng Zhou, Xue-Yan Kui, Xiao-Hui Li, Chang-Bin Ke, Zhi-Jun Pei

**Affiliations:** 1 Postgraduate Training Basement of Jinzhou Medical University, Taihe Hospital, Hubei University of Medicine, Shiyan, Hubei, China.; 2 Department of Nuclear Medicine and Institute of Anesthesiology and Pain, Taihe Hospital, Hubei University of Medicine, Shiyan, Hubei, China.; 3 Department of Pathology, Taihe Hospital, Hubei University of Medicine, Shiyan, Hubei, China.; 4 Hubei Key Laboratory of Embryonic Stem Cell Research, Shiyan, Hubei, China.; 5 Hubei Key Laboratory of WudangLocal Chinese Medicine Research, Shiyan, Hubei, China.; 6 Department of Radiology, Xiangyang Central Hospital, Affiliated Hospital of Hubei University of Arts and Science, Xiangyang, Hubei, China.

**Keywords:** Colorectal cancer, EIF2S2, ^18^F-FDG, SUVmax, glucose transporter 1

## Abstract

**Background:** We sought to investigate whether the expression of the gene EIF2S2 is related to ^18^F-FDG PET/CT metabolic parameters in patients with colorectal cancer (CRC).

**Materials and methods:** The expression of EIF2S2 in CRC and its relationship with clinicopathological features were obtained through the ONCOMINE, UALCAN and GEPIA databases. EIF2S2 and GLUT1 expression were examined by immunohistochemistry in 42 CRC patients undergoing preoperative PET-CT examination. Spearman correlation analysis was used to assess the relationship between EIF2S2 and GLUT1 levels and clinical parameters. Correlation analysis between EIF2S2 and Reactome-Glycolysis signatures was performed using GEPIA2. We describe the effect of EIF2S2 knockdown on lactate production and the mRNA levels of glycolysis-related genes in human colon cancer SW480 cells.

**Results:** Immunohistochemistry revealed an upregulation of EIF2S2 protein expression in tumor tissues of colorectal cancer patients, which is consistent with the significant upregulation of EIF2S2 transcript levels in the database. These colorectal cancer patients included 24 cases of colon cancer and 18 cases of rectal cancer, ranging in age from 31 to 78 years. The transcription was significantly related to histological subtypes and TP53 mutations (P <0.05). The value of SUVmax in CRC significantly correlated with the expression of EIF2S2 (rho = 0.462, P <0.01). Although SUVmax and SUVmean was not correlate with the expression of GLUT1 (P <0.05), a significant correlation was observed between the expression of GLUT1 and the volumetric PET parameters, such as MTV and TLG (*P* < 0.01). GLUT1 expression in CRC was positively correlated with EIF2S2 status (rho = 0.470, P <0.01). In SW480 cells, RNAi-mediated depletion of EIF2S2 inhibited lactic acid production (P <0.05) and SLC2A1, SLC2A3, SLC2A10, HK2, PKM2, LDHA mRNA level (P <0.01).

**Conclusions:** Primary CRC FDG uptake is strongly associated with the overexpression of EIF2S2, and EIF2S2 may promote glycolysis in CRC by mediating GLUT1.

## Introduction

Colorectal cancer (CRC) is the fourth most common cancer 1. Although the incidence and mortality of CRC have declined over the past two decades, unresectable locally advanced CRC has a poor prognosis. Early diagnosis and timely surgical treatment can significantly reduce the mortality of patients. There is a need for effective biomarkers of CRC for screening and early detection. Cancer cells mainly obtain energy by breaking down glucose into ATP using aerobic glycolysis (the Warburg effect) 2, 3. ^18^F-FDG PET/CT has been widely used in the detection, treatment monitoring, and prognostic evaluation of various tumors 4-7. ^18^F-FDG is glucose radiolabeled with ^18^F. Drawn in by glucose transporters (GLUTs) and phosphorylated by hexokinases (HK), it cannot be metabolized further, allowing its irreversible entry into cells. ^18^F-FDG is used to detect the amount of glucose uptake in tumors. The mechanism may be mediated by the increased number of GLUTs 8 and HK 9, 10 in malignant cells. The relationship between expression of GLUT-1 or HK2 and degree of ^18^F-FDG uptake has been extensively studied 11-16. High expression of GLUT-1 is a crucial factor for ^18^F-FDG uptake in many malignant tumors. GLUT1 is encoded by the SLC2A1 gene and plays an important role in the development of many tumors, such as nonsmall cell lung cancer 17 and breast cancer 18. In previous reports, it was found that the relationship between ^18^F-FDG uptake and GLUT-1 expression in different malignant tumors is not entirely consistent 19, 20. It has been reported that ^18^F-FDG uptake correlates with GLUT1 expression in patients with liver metastasis from CRC 21. In addition, it showed that SUVmax may be useful for predicting the NPM1 expression of lung adenocarcinoma 22 and METTL3 expression of esophageal cancer 23.

RNA binding protein (RBP) is widely recognized as a protein that plays an important role in regulating gene expression 24. At present, more and more RBPs have been discovered, which play an important role in promoting the occurrence and development of tumors 25. Some RBPs can also mediate energy metabolism 26. EIF2 is a heterotrimer consisting of eIF2*α*, eIF2*β* and eIF2*γ* 27, 28. EIF2S2 (Eukaryotic Translation.

Initiation Factor 2 Subunit *β*) showed a significant relationship between DNA copy number levels and mRNA expression in luminal breast tumors 29. The eIF2*β* gene is highly expressed in lung cancer and can indicate the prognosis of patients with lung adenocarcinoma 30. EIF2S2 may play a role in promoting tumors by regulating the WNT signaling pathway 31. But whether EIF2S2 mediates aerobic glycolysis has not been reported in CRC.

Transcriptional data for EIF2S2 in CRC and its clinically relevant data were obtained from The Cancer Genome Atlas (TCGA) database. We confirmed the high expression of EIF2S2 in CRC through immunohistochemistry. The correlation between EIF2S2 and 18F-FDG uptake in colorectal cancer and the effect of EIF2S2 knockdown on the levels of genes related to lactate production and partial glycolysis in SW480 cells were investigated.

## Methods

### ONCOMINE database analysis

The ONCOMINE database (www.oncomine.org) is an integrated online cancer microarray database for DNA or RNA sequence analysis, designed to facilitate discoveries from whole gene expression analysis^32^. In this study, using the ONCOMINE database, we obtained data on the transcriptional expression differences of EIF2 between CRC and normal colorectal tissues. The cutoff values were as follows: *P* value: 1E-4; fold change: 2; gene rank: 10%; data type: mRNA.

### UALCAN

UALCAN (http://ualcan.path.uab.edu) is a comprehensive, user-friendly, and interactive web resource for analyzing cancer omics data from the TCGA database 33. Differences in EIF2S2 transcript levels were identified through the UALCAN website, including 286 colon cancer tissues and 41 normal colon tissues; 166 rectal cancer tissues and normal rectal tissues in the TCGA database. At the same time, we analyzed the relative expression of EIF2S2 in different groups with variations in sex, age, tumor histology, tumor grade, tumor stage, and lymphnode status). *P* < 0.05 was considered statistically significant.

### GEPIA

Gene Expression Profiling Interactive Analysis (http://gepia.cancer-pku.cn/index.html) is a tool to analyze RNA sequence expression data of 9736 tumors and 8587 normal tissues 34. We found 83 Reactome-Glycolysis signaling molecules from the Reactome database 35. The correlation between EIF2S2 and glycolysis and glucose transport-related proteins was analyzed.

### Study population

The study included 42 patients with CRC undergoing ^18^F-FDG PET/CT examination and surgical resection from February 2015 to October 2019. Inclusion criteria were as follows: (a) CRC confirmed by pathology; (b) no biopsy, radiotherapy or chemotherapy before PET/CT; (c) surgery was performed within 4 weeks after the scan; (d) tissue samples were available for immunohistochemistry (IHC) staining; and (e) case records were complete. The age range of the 28 male patients was 31-78 with a mean age of 57.5 years. 14 female patients had an age range of 40-80 years with a mean age of 57.7 years. They were pathologically diagnosed with CRC and did not receive radiotherapy or chemotherapy before surgical resection and PET/CT imaging.

### ^18^F-FDG PET/CT

PET/CT images (Biograph MCT; Siemens) were acquired 1 h after intravenous injection of ^18^F-FDG and fasting for at least 6 h. Regions of interest (ROIs) were drawn around the contours of the primary tumors on the PET images. SUVmax, SUVmean, total lesion glycolysis (TLG), and metabolic tumor volume (MTV) of each primary tumor were automatically calculated and recorded.

### Immunohistochemistry and analysis

After the CRCs were resected, the tumor tissues and adjacent tissues were subjected to immunohistochemical analysis of the expression of EIF2S2 and GLUT1. The sections were deparaffinized, rehydrated, and incubated with citrate buffer under high pressure for 3 min. After cooling to room temperature, the slices were placed in 3% hydrogen peroxide and incubated for 12 min. After blocking the sections with the serum for 20 min, they were incubated with anti-EIF2S2 antibody (1:400, Abcam) and anti-GLUT1 antibody (1:200, Abcam) overnight at 4 °C. The slices were cleaned with tris-buffered saline Tween-20. Donkey anti-rabbit (1:300, Abcam) and goat anti-mouse (1:400, Abcam) secondary antibodies were dropped onto the slice to cover the tissues and then incubated at room temperature for 50 minutes. After the sections were cleaned with TBST, diaminobenzaldehyde (DAB) reagent was added dropwise to develop the color. After rinsing, they were stained with hematoxylin. Next, the slices were dehydrated and sealed. Two experienced physicians evaluated all immunohistochemical staining results and reached agreement. The intensity of membrane/cytoplasmic staining was scored as follows: strong staining of >50% of cancer cells, 3+; moderate staining of ≥10%-50% of cancer cells, 2+; weak staining of <10% of cancer cells, 1+; and no staining, 0. Cases with scores of 2+ and 3+ were rated as highly positive.

### Cell culture and cell transfection

Colon cancer cell line SW480 was purchased from the cell bank of the Shanghai Institute of Life Sciences. SW480 was cultured in H-DMEM (GIBCO) supplemented with 10% fetal bovine serum and maintained at 37 °C and 5% CO2 in humid air. EIF2S2 siRNA and a negative control siRNA were designed and synthesized by the Shanghai Gene Pharmaceutical Company. siRNA sequences were listed in Table A .

On the day before transfection, approximately 2 × 10^6^ cells per well were inoculated into 6-well plates. According to the manufacturer's instructions, siRNA was transfected with Lipofectamine™ Reagent (Invitrogen).

### Quantitative real-time PCR assay

SW480 cells in the logarithmic growth phase were inoculated with 1 × 10^5^ cells in 24-well plates and infected with siEIF2S2 or siCtrl. Total RNA was extracted from cells and tissues with Trizol reagent (Invitrogen) and a real-time polymerase chain reaction (RT-PCR) was performed with PrimeScript™ RT Master Mix (TakaRa). The expression level of the target gene was analyzed by qRT-PCR in SYBR Green qPCR main mixed reagent system (TakaRa). The PCR primers used are listed in Table B.

### Lactate production assays

An L-Lactic Acid Colorimetric Assay Kit (Elabscience) was used to measure the lactate production according to the manufacturer's protocols. The transfected cells were seeded into 96-well cell culture plate and incubated at 37 °C overnight. After starvation for 2 h, the supernatant was collected to determine lactic acid production. Lactate production was measured at 530 nm with a microplate reader.

### Statistical analyses

The expression of EIF2S2 and GLUT1 in tumor tissues and adjacent normal tissues was analyzed by an independent sample t-test. The chi-squared test was used to analyze the correlation between EIF2S2 expression and clinical parameters in patients with CRC. Spearman's correlation coefficient was used for correlation analysis among SUVmax, EIF2S2, and GLUT1 expression. The ROC curve was used to analyze the accuracy of SUVmax to predict the expression of EIF2S2. Multivariate analysis was used to analyze the factors related to the expression of EIF2S2. P <0.05 was considered significant. SPSS software (SPSS, version 26.0) was used for statistical analysis.

## Results

### Overexpression of EIF2S2 gene in CRC

**Figure 1:**
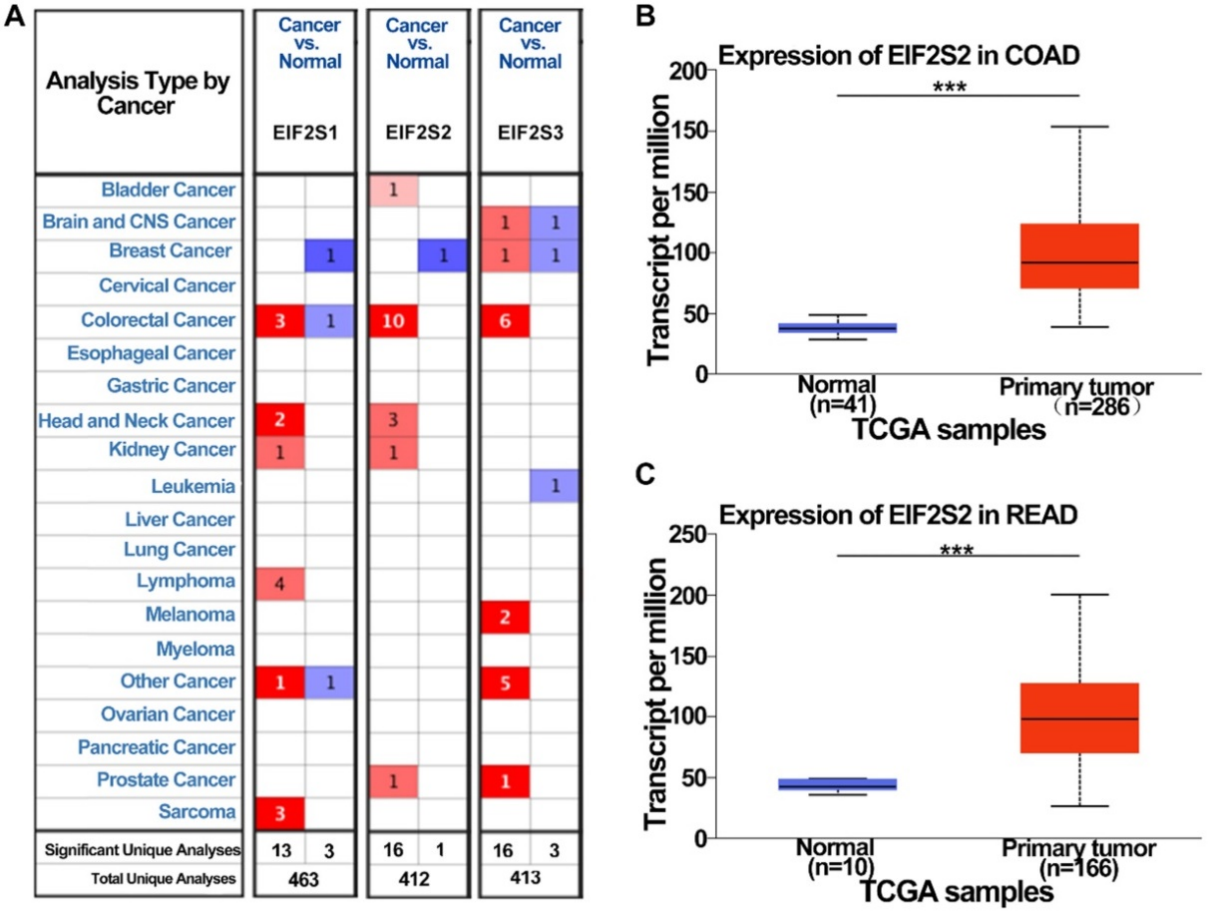
The expression level of EIF2S2 in CRC and adjacent normal tissues and from the TCGA. **A,** EIF2S2-EIF2S3 mRNA expression (cancer tissue VS normal tissue) was analyzed using the Oncomine database. The numbers in colored cells show the quantities of datasets with statistically significant mRNA overexpression (red) or underexpression (blue) of target genes. Cell color was determined by the best gene rank percentile for the analysis within the cells. The number in each cell represents the number of analyses that satisfied the threshold, such as gene rank percentile (10%), P-value (1E-4), and fold change (2). **B,** Comparison of EIF2S2 expression between COAD and normal colon tissue. **C,** Comparison of EIF2S2 expression between READ and normal colon tissue. ***P<0.001.

To examine the potential role of EIF2s in CRC development, we first analyzed the expression of EIF2s in normal samples and CRC tissues with the Oncomine database. We found that the expression of EIF2S1, EIF2S2, and EIF2S3 in CRC was higher than that in normal colorectal tissues in several databases (Fig. 1A, *P* < 0.001). The transcription rate of EIF2S2 in CRC tissues was significantly higher than that in normal colorectal tissues by the UALCAN database (Fig. 1B, C; *P* < 0.001). These results suggest that EIF2S2 may promote tumorigenesis and progression of CRC.

### Relationship between transcriptional rate of EIF2S2 and clinical data of CRC patients

**Figure 2:**
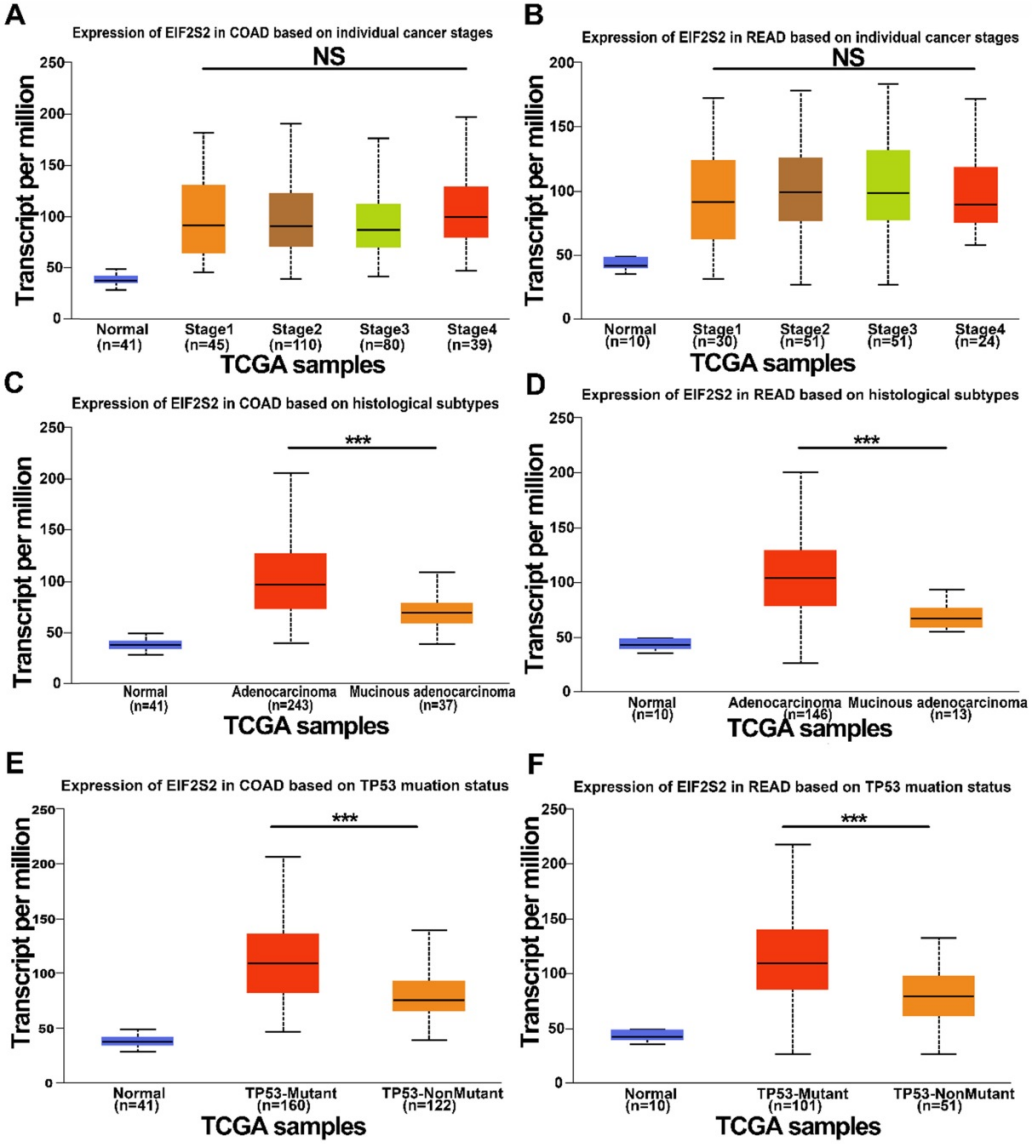
The expression level of EIF2S2 in CRC and adjacent normal tissues from the TCGA. **A,** The expression of EIF2S2 in COAD based on different individual cancer stages VS normal tissues by RNA-Seq derived expression data. **B,** The expression of EIF2S2 in READ, based on different individual cancer stages VS normal tissues by RNA-Seq derived expression data. **C,** The expression of EIF2S2 in COAD based on different histological subtypes VS normal tissues by RNA-Seq derived expression data. **D,** The expression of EIF2S2 in READ based on different histological subtypes VS normal tissues by RNA-Seq derived expression data. **E,** The expression of EIF2S2 in COAD based on different TP53 mutation status VS normal tissues by RNA-Seq derived expression data. **F,** The expression of EIF2S2 in READ based on different TP53 mutation status VS normal tissues by RNA-Seq derived expression data. ***P<0.001.

We further examined the expression of EIF2S2 expression in human CRC in the TCGA data using the UALCAN database. It revealed that EIF2S2 remained the same regardless of sex, age, or node metastasis status in CRC patients. Surprisingly, the expression of EIF2S2 had no significant correlation with tumor stage (Fig. 2A, B; *P* >0.05). However, EIF2S2 expression in colorectal cancer was significantly correlated with TP53 mutation status and tumor histological subtype (Fig. 2C-F [*P* < 0.001]).

### Patient characteristics

The clinical data of the 42 patients with CRC was shown in Table 1. There were 28 males and 14 females. There were 21 patients aged ≥ 60 y and 21 patients < 60 y. A total of 24 cases occurred in the colon, and 18 cases occurred in the rectum. There were 15 tumors ≤ 3 cm and 27 > 3 cm. The degree of differentiation of tumors was divided into 4 types: 3 patients with poorly differentiated, 11 patients with poorly-moderately differentiated, 25 patients with moderately differentiated, and 3 patients with well-differentiated tumors. The tumor stages were 4 patients with Stage 1, 15 with Stage 2, 22 with Stage 3, and 1 with Stage 4. Further analysis of clinical data revealed that the mean SUVmax of the CRCs was 22.4, ranging from 6.88-56.66 (Table 2).

### Expression of EIF2S2 in CRC patients and its relationship to clinical features

**Figure 3:**
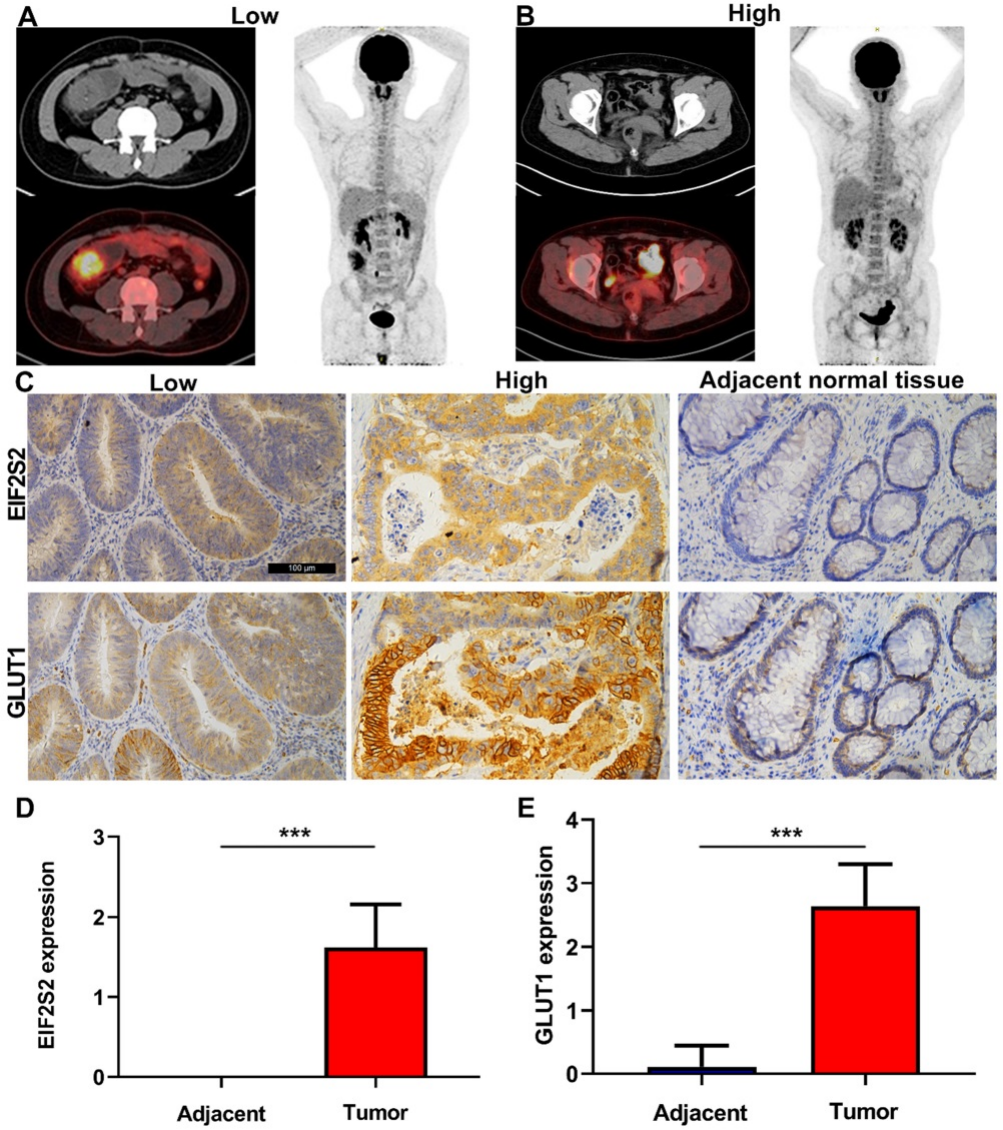
Representative images of PET/CT and immunohistochemistry. **A,** PET/CT imaging for CRC patient with high SUVmax (left) and low SUVmax (right). **B,** Immunohistochemical staining for EIF2S2 and GLUT1 in CRC tissue with different SUVmax and adjacent normal lung tissues (magnification, ×100). **C,** The mean immunohistochemical staining levels of EIF2S2 (1.96 ± 0.84) in CRC tissue was significantly higher than that of matched adjacent normal lung tissue (0.13 ± 0.34). **D,** The mean immunohistochemical staining levels of GLUT1 (1.65 ± 0.90) in CRC tissue was significantly higher than that of matched adjacent normal Colorectal tissue (0.83 ± 0.38). ***P<0.001.

The SUVmax values of tumors in CRC patients were higher than those in normal tissues, but varied (Fig. 3A). Through immunohistochemical staining of the tissues of 42 patients with CRC, we found that EIF2S2 and GLUT1 were more highly expressed in tumor tissues than in adjacent normal tissues. In addition, we found that EIF2S2 is mainly expressed in the cytoplasm, while GLUT1 is expressed in both the cytoplasm and the cell membrane (Fig. 3B). The positive rate of EIF2S2 expression in tumor tissues is 97.6% (41/42) and 0% (0/10) in adjacent normal tissues. Positive GLUT1 expression was observed in 100% (42/42) of the tumors and 10% (1/10) of adjacent normal tissues. The average staining intensity scores of EIF2S2 and GLUT1 in tumor tissues were significantly higher than those in adjacent normal tissues (Fig. 3C, D; *P* <0.001). Multivariate analysis demonstrated there were no significant differences between EIF2S2 expression and sex, age, tumor size, histological differentiation, tumor staging and microsatellite stability immunohistochemical scores (*P* > 0.05). However, there was a significant correlation between SUVmax and EIF2S2 expression (Tables 3 & 4).

### SUVmax positively correlated with the expression of EIF2S2

**Figure 4:**
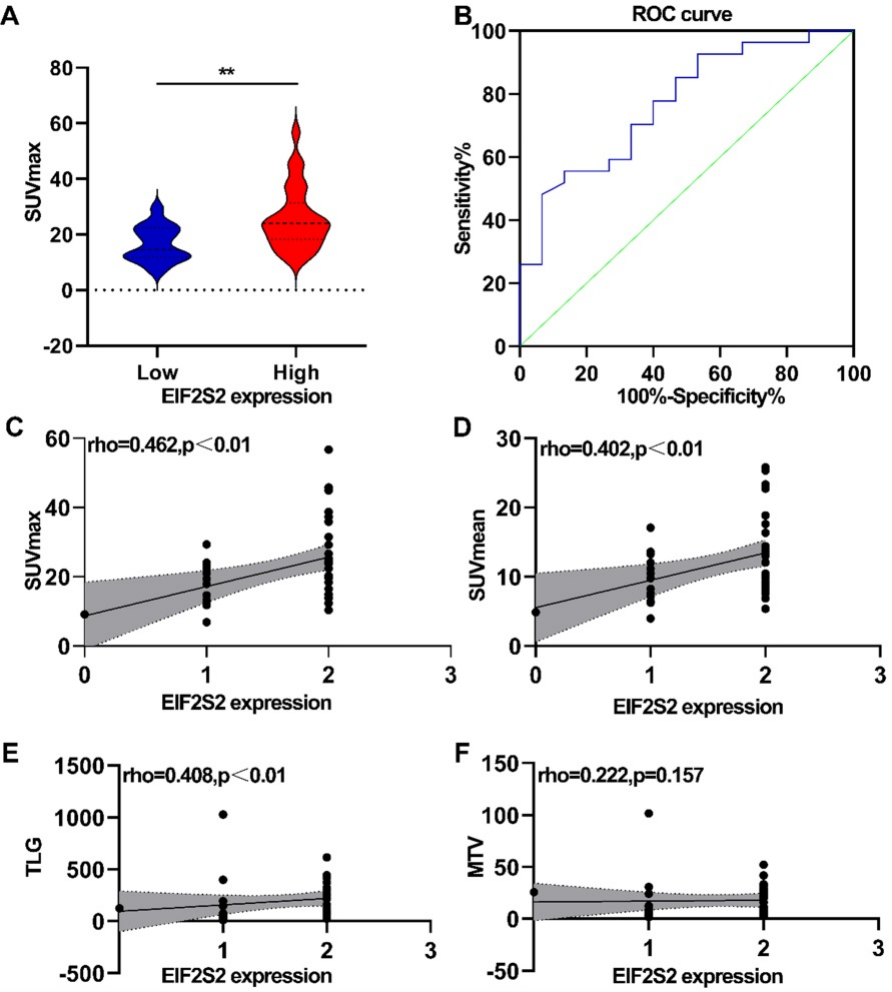
Correlation analysis between PET/CT metabolic parameters and IHC score. **A,** The SUVmax was higher in high expression of EIF2S2.**B,** Determination of the cutoff value of SUVmax by the receiver operating characteristics (ROC) curve. The ROC curve was used to determine the optimal cutoff value of SUVmax for predicting EIF2S2 high positive CRC. Area under the curve: 0.769; 95% CI: 0.623 to 0.915. **C, D** The SUVmax, SUVmean TLG showed a linear correlation with the EIF2S2 IHC score with a correlation coefficient of 0.462, 0.402, 0.408respectively (P< 0.001). E, F, There was no significant correlation between EIF2S2 IHC score and MTV. **P<0.01.

The correlation between PET/CT metabolic parameters and EIF2S2 expression in CRC was analyzed. In CRC, tumor tissues with high expression of EIF2S2 had a significantly higher SUVmax than tissues with low expression (25.6 ± 11.29 vs 16.56 ± 6.39) (Fig. 4A, *P* <0.05). The ROC curve analysis showed that the area under the curve was 0.769 ± 0.090 (95% CI = 0.623-0.915, *P* = 0.0042) (Fig. 4B). The expression rate of EIF2S2 in CRC patients was significantly positively correlated with SUVmax, SUVmean, and TLG (Figure 4C-E; *P* < 0.01), but not with MTV (Fig. 4F, *P* >0.05). These results suggest that the SUVmax of the tumor in patients with CRC has a certain predictive value for the expression of EIF2S2.

### Correlation between the parameters of ^18^F-FDG uptake and GLUT1 expression

By analyzing the relationship between PET/CT parameters and GLUT1 expression, no significant correlation was detected between SUVmean and SUVmax in ^18^F-FDG and GLUT1 expression (*P* > 0.05). In contrast, TLG and MTV were significantly correlated with GLUT1 expression (*P* < 0.01) (Table 5).

### EIF2S2 may mediate glycolytic metabolism in CRC patients

**Figure 5:**
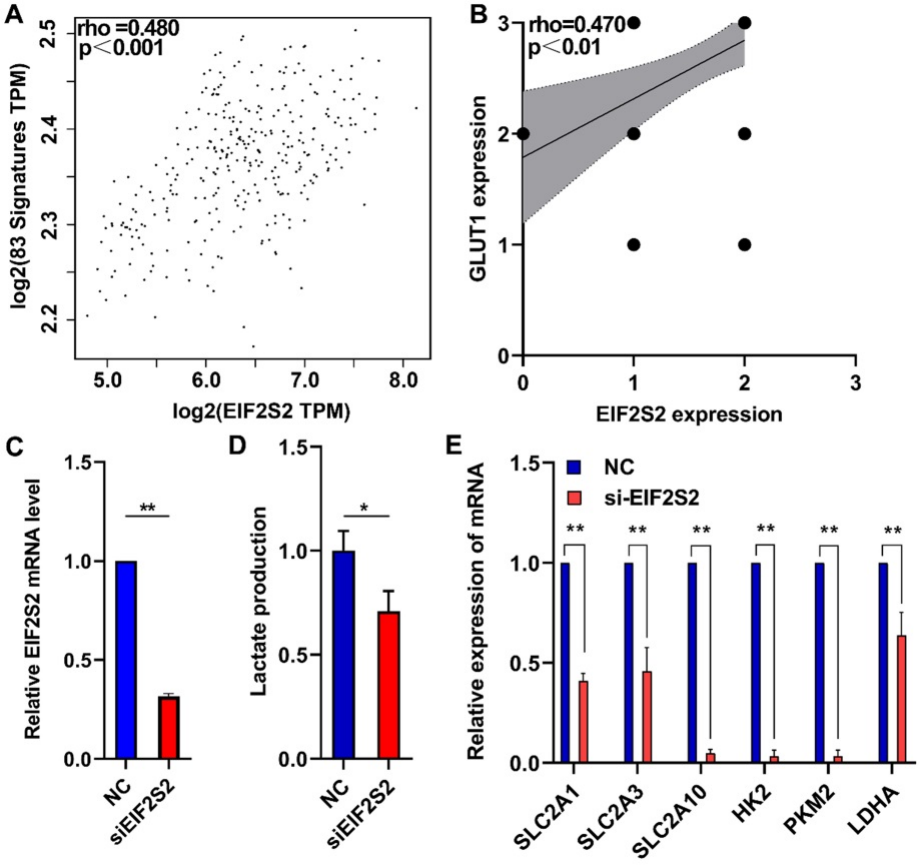
EIF2S2 is closely correlated with glycolytic metabolism in CRC. **A,** The expression of EIF2S2 showed a linear correlation with related to Reactome-Glycolysis and Reactome-Glucose-Transport gene sets with a correlation coefficient of 0.480, respectively (P<0.001) . **B,** The expression of EIF2S2 showed a linear correlation with the expression of GLUT1 with a correlation coefficient of 0.470 in CRC, respectively (P<0.01). **C,** Real-time PCR assays were performed to analyze the relative EIF2S2 levels in SW480 cells after knockdown of EIF2S2, n=3. **D,** Lactate production were assessed in SW480 cells after knockdown of EIF2S2, n= 3 **E,** Real-time PCR assays were performed to analyze the relative SLC2A1, SLC2A3, SLC2A10,HK2,PKM2,LDHA levels and in SW480 cells after knockdown of EIF2S2, n=3. * P<0.05, ** P<0.01.

A total of 83 glycolysis and glucose transporters were found from the Reactome database. A significant correlation was found between glycolysis signal molecules and EIF2S2 by using the GEPIA database (Fig. 5A). From the analysis of the previous immunohistochemistry results, there was a significant positive correlation between the expression of EIF2S2 and GLUT1 (Fig. 5B). Further lactate production assays showed that lactic acid production (a key metabolite of glycolysis) was significantly decreased after EIF2S2 knockdown in SW480 cells (Fig. 5C, D). SLC2A1, SLC2A3, SLC2A10, HK2, PKM2, and LDHA were significantly decreased after EIF2S2 knockdown in SW480 cells (Fig. 5E). These data indicate that EIF2S2 may mediate glycolysis in CRC, thereby promoting the occurrence and development of CRC.

## Discussion

In this paper, to study the expression of EIF2S2 in CRC comprehensively, the overexpression of EIF2S2 and its relationship with clinicopathological features were identified using the TCGA database. The results showed that the expression of EIF2S2 was significantly higher in CRC than adjacent normal tissues, the higher expression of EIF2S2 is associated with worse overall survival of patients with CRC 31. Therefore, EIF2S2 is expected to be a prognostic marker and potential therapeutic target for CRC.

It was found that EIF2S2 expression is associated with tissue subtype and TP53 mutation, but not related to age, sex, tumor differentiation, tumor stage, or lymphnodal status in CRC patients. We performed immunohistochemistry on the surgically removed tumor tissues and adjacent normal tissues in 42 patients with CRC. That EIF2S2 expression is not related to age, sex, tumor differentiation, or tumor stage, is consistent with the data from the UALCAN database. When we analyzed the correlation between EIF2S2 and Mismatch Repair (MMR) protein expression, it was found that there was no significant correlation between the expression of EIF2S2 and the expression of MSH2 and PMS2. But there are no patients with microsatellite instability in 31 patients, the relationship between EIF2S2 and microsatellite stability could not be evaluated. By analyzing the correlation between the expression level of EIF2S2 in CRC patients and the related parameters of PET/CT (SUVmax, SUVmean, MTV, and TLG), we found that CRC with high EIF2S2 expression is accompanied by high uptake of ^18^F-FDG. ROC analysis showed that ^18^F-FDG uptake could predict the expression of EIF2S2 in CRC patients. In addition, multivariate analysis showed that there was a correlation between SUVmax and EIF2S2 expression. This result suggests that we can predict the expression of EIF2S2 through SUVmax, and also suggests that EIF2S2 may affect the glucose uptake process of aerobic glycolysis in CRC.

Immunohistochemical staining confirmed the correlation between EIF2S2 expression and GLUT1 expression. We confirmed that some glycolysis-related mRNA levels and lactate production and some glycolysis-related mRNA levels (SLC2A1, SLC2A3, SLC2A10, HK2, PKM2, LDHA) were reduced after EIF2S2 knockdown. Therefore, these results suggest that EIF2S2 may be involved in the glycolysis by regulating GLUT1 in CRC. In previous reports, there have been different opinions about SUVmax and GLUT1 in CRC 36, 37. Moreover, the values of MTV and TLG have been linked with the prognosis of CRC 38, 39. While TLG and MTV significantly correlated with GLUT1, the correlation between SUVmax, SUVmean and the expression of GLUT1 had no significant correlation in the study. Although EIF2S2 was significantly correlated with SUVmax and GLUT1 in CRC, it is not known whether EIF2S2 affects glucose uptake by acting directly on GLUT1.

The TCGA database shows that the expression of EIF2S2 is related to the mutation level of TP53 in CRC. The p53 pathway plays a vital role in the aerobic glycolysis pathway of malignant tumor 40, 41. Jiwei Zhang et al. reported that EIF2S2 could interact with c-MYC 31. In tumor cells, c-MYC can promote the expression of glucose transporter 1 (GLUT1) 42. We speculate that EIF2S2 may regulate the metabolism of CRC by inhibiting the p53 signaling pathway or promoting the c-MYC-GLUT1 signaling pathway. In the future, we plan to analyze how EIF2S2 acts on the anaerobic glycolysis mechanism of CRC.

The present study is not without limitations. First of all, due to geographical limitations and the absence of multicenter sampling, the samples in this study are not representative of the whole population, which increases the possibility of selection bias. Second, our study sample size was relatively small. Third, our study was retrospective in nature. Therefore, a larger sample of cases, multicenter sampling, and prospective randomized studies are needed to verify our findings.

We tentatively put forward that the common PET/CT parameter SUVmax has a certain predictive value for the expression of EIF2S2 in CRC. Meanwhile, the comprehensive analysis of the TCGA database and clinicopathological features including PET parameters not only provides us with new ideas for finding new diagnostic and therapeutic targets for tumors, but also provides a theoretical basis for us to study further the pathways of differential gene expression affecting tumor aerobic glycolysis analysis.

## Figures and Tables

**Table A:** List of siRNA sequences

Name	Sequence
siEIF2S2	Forward 5'- GUCGUCCGAGUAGGAACCATT-3'
	Reverse 5'- UGGUUCCUACUCGGACGACUU-3'
NC siRNA	Forward 5'- UUCUCCGAACGUACGUTT-3'
	Reverse 5'- ACGUGACACGUUCGGAGAATT-3'

**Table B:** List of PCR primers

Gene name	Primer sequence
EIF2S2	Forward 5'- ACACATACGAGGAGCTGCTGAATC-3'
	Reverse 5'- AG CTTGGTTCCTACTCGGACGACTTG-3'
SLC2A1	Forward 5'- TGTCTGGCATCAACGCTGTCTTC-3'
	Reverse 5'- TC CCTGCTCGCTCCACCACAAAC-3'
SLC2A3	Forward 5'- TCAATGTGCAGTGTAGCCCA-3'
	Reverse 5'- AG CTGCCTTACTGCCAACCTAC-3'
SLC2A10	Forward 5'- TCATTGGCACCATCGGCTTGTC-3'
	Reverse 5'- CC GGTGAACCGTCTCTTCTGGAACTG-3'
HK2	Forward 5'- CGACAGCATCATTGTTAAGGAG-3'
	Reverse 5'- CA GCAGGAAAGACACATCACATTT-3'
PKM2	Forward 5'- TGCCGCCTGGACATTGATTCAC-3'
	Reverse 5'- GA GTTCAGACGAGCCACATTCATTCC-3'
LDHA	Forward 5'- TGGCAACTCTAAAGGATCAGC-3'
	Reverse 5'- TA CCAACCCCAACAACTGTAATCT-3'
β-actin	Forward 5'- GGAGA TTACTGCCCTGGCTCCTA-3'
	Reverse 5'- GACTCA TCGTACTCCTGCTTG-CTG-3'

**Table 1:** Clinicopathological characteristics of 42 patients

Variables	N	EIF2S2 expression			GLUT1 expression		
		Low	High	*P*-value	Low	High	*P*-value
**Sex**							
Male	28	11	17	0.506	3	25	0.718
Female	14	4	10		1	13	
**Age**							
<60 years	21	10	11	0.113	2	19	1
≥60 years	21	5	16		2	19	
**Size**							
≤3 cm	15	8	7	0.079	2	13	0.542
>3 cm	27	7	20		2	25	
**Primary lesion**							
Colon	24	8	16	0.718	3	21	0.460
Rectum	18	7	11		1	17	
**Histological differentiation**							
Poorly	3	2	1	0.826	0	3	0.079
Poorly-Moderately	11	3	8		0	11	
Moderately	25	9	16		3	22	
Well	3	1	2		1	2	
**Tumor staging**							
1	4	1	3	0.455	1	3	0.708
2	15	5	10		0	15	
3	22	8	14		3	19	
4	1	1	0		0	1	

**Table 2:** Median values of PET parameters (min-max)

PET metabolic parameter	Value
SUVmax	22.4 (6.88-56.66)
SUVmean	11.9 (3.96-25.81)
TLG	194.7 (9.47-1025.46)
MTV	17.5 (1.56-101.4)

**Table 3:** Multivariate analysis of EIF2S2 expression in 42 CRC patients

Parameter	Univariate analysis		P
	OR	95%CI	
Sex	1.997	0.409-9.740	0.393
Age (years)	2.472	0.610-10.025	0.205
Tumor size (cm)	3.110	0.760-12.722	0.115
Histological differentiation	1.111	0.437-2.825	0.825
Tumor staging	0.599	0.212-1.689	0.333
SUVmax	5.000	1.143-21.864	0.033

OR: Odds ratio. 95% CI: 95% Confidence interval.

**Table 4:** Comparison of STR IHC score with EIF2S2 expression

Variables	N	EIF2S2 expression		
		Low	High	*P*-value
**MSH1**				
Low	2	0	2	0.203
High	29	10	19	
**MSH2**				
Low	0	0	0	
High	31	10	21	
**MSH6**				
Low	0	0	0	
High	31	10	21	
**PMS2**				
Low	8	2	6	0.605
High	23	8	15	

**Table 5:** Comparison of PET parameter according to GLUT1 expression

PET metabolic parameter	GLUT1-Low (*n* = 11)	GLUT1-High (*n* = 31)	*P*-value
SUV_max_ (mean ± SD)	25.02 ±14.06	22.14 ±10.48	0.714
SUV_mean_ (mean ± SD)	13.99± 8.18	11.71 ±5.09	0.620
TLG (mean ± SD)	73.64±51.14	207.47± 202.89	0.005
MTV (mean ± SD)	6.22± 4.37	18.70± 18.36	0.003

## Abbreviations

CRC: Colorectal carcinoma
COAD: colon adenocarcinoma
READ: rectal adenocarcinoma
EIF2S2: Eukaryotic Translation Initiation Factor 2 Subunit Beta
PET/CT: Positron emission tomography/computed tomography
^18^F-FDG: ^18^F-fluorodeoxyglucose
SUV: standard uptake value
GLUT1: Glucose transporter 1
HK2: hexokinase 2
TCGA: The Cancer Genome Atlas
ROI: region of interest
TBST: Tris-Buffered Saline Tween-20
SD: standard deviation
ROC: receiver operating characteristic
